# Protective effects of miR-29a on diabetic glomerular dysfunction by modulation of DKK1/Wnt/β-catenin signaling

**DOI:** 10.1038/srep30575

**Published:** 2016-07-27

**Authors:** Yung-Chien Hsu, Pey-Jium Chang, Cheng Ho, Yu-Ting Huang, Ya-Hsueh Shih, Ching-Jen Wang, Chun-Liang Lin

**Affiliations:** 1 Departments of Nephrology, Chang Gung Memorial Hospital, Chiayi, Taiwan; 2 Kidney Research Center, Chang Gung Memorial Hospital, Taipei, Taiwan; 3 Colleage of Medicine, Chang Gung University, Taiwan; 4 Graduate Institute of Clinical Medical Sciences, Chang Gung University, Taiwan; 5 Division of Endocrinology and Metabolism, Chang Gung Memorial Hospital, Chiayi, Taiwan; 6 Center for Shockwave Medicine and Tissue Engineering, Department of Medical Research, Chang Gung Memorial Hospital, Kaohsiung, Taiwan; 7 Department of Orthopedic Surgery, Chang Gung Memorial Hospital, Kaohsiung, Taiwan; 8 Kidney and Diabetic Complications Research Team (KDCRT), Chang Gung Memorial Hospital, Chiayi, Taiwan

## Abstract

Dysregulation of specific microRNAs or Wnt/β-catenin signaling pathway is critically implicated in the pathogenesis of various renal diseases. However, the relationship between microRNAs and Wnt/β-catenin signaling in diabetes-induced glomerular sclerosis remains unknown. Here, we found that decreased miR-29a expression and attenuated Wnt/β-catenin signaling were concomitantly detected in glomeruli of streptozotocin-induced diabetic mice. Gain of miR-29a function in diabetic mice substantially increased the expression of β-catenin and blocked the expressions of profibrotic gene markers, including DKK1 (a Wnt antagonist), TGF-β1 and fibronectin, in glomerular mesangium. Moreover, in the normal mice treated with miR-29a inhibitor, renal fibrosis was induced with an attenuated Wnt/β-catenin signaling activity. Consistently, the constructed miR-29a transgenic mice that supported sustained Wnt/β-catenin signaling had the ability to block the expressions of profibrotic genes after induction of diabetes. We also demonstrated that miR-29a acts as a positive regulator of Wnt/β-catenin signaling in cultured mesangial cells and functions to protect cell apoptosis and fibrosis. Importantly, we showed that activation of Wnt/β-catenin signaling in cultured mesangial cells by transfecting the β-catenin (Δ45) mutant or by a GSK-3β inhibitor reversely upregulated miR29a. Our findings suggest that the reciprocal relationship between miR-29a and DKK1/Wnt/β-catenin signaling may play an important part in protecting renal fibrogenesis.

Diabetes-mediated kidney disorders are common etiological causes of end-stage renal disease^1^. During the progression of diabetic nephropathy, the most prominent pathological feature is glomerulosclerosis, which is caused by excessive mesangium remodeling^2^. With time, intensive fibrosis reactions induced by hyperglycemia in glomerular mesangium, including increased levels of inflammatory and profibrotic mediators such as TGF-β1, as well as increased accumulation of extracellular matrix proteins such as fibronectin and collagens, ultimately lead to diabetes-induced renal failure^3^,^4^,^5^. Loss of mesangial cell viability is also reported as an important pathological event in the diabetic renal tissue destruction^6^,^7^.

The molecular mechanisms that promote diabetic glomerulosclerosis are necessarily complex and multifactorial. Although numerous signaling pathways or components that promote deposition of extracellular matrix proteins have been increasingly reported in different *in vitro* or *in vivo* diabetic models^8^,^9^,^10^,^11^,^12^, substantial interplay or cross talk of these identified signaling components still remains to be established. Previously, we have demonstrated that attenuated Wnt/β-catenin signaling is closely associated with the development and progression of renal fibrosis in cell culture and animal models^9^. We later found that Dickkopf-1 (DKK1), a Wnt antagonist, plays a critical role in the attenuation of the Wnt/β-catenin signaling activity in renal mesangial cells under high glucose conditions^10^. Treatment of diabetic rats with DKK1 antisense oligonucleotide significantly alleviated hyperglycemia-induced excretion of urinary proteins, and hyperglycemia-promoted upregulation of TGF-β1 and fibronectin in glomerular mesangium^10^. Currently, very little is known about the upstream regulation of DKK1 and Wnt/β-catenin signaling in diabetes-induced renal fibrosis.

MicroRNAs are short non-coding RNAs that repress protein synthesis through mRNA degradation or translational inhibition^13^. Growing evidence has shown that microRNAs are involved in modulation of cell survival and differentiation^14^, as well as tissue metabolism and remodeling^15^. In kidneys, microRNAs are not only implicated in renal development and homeostasis, but also play important roles in the pathogenesis of renal diseases including renal fibrosis^16^,^17^. Upregulation of specific microRNAs, including miR-192, miR-200b/c, miR-217, miR-216a and miR-377, has been reported to promote glomerular fibrosis and hypertrophy in various animal models^16^,^17^. Among these microRNAs, miR-192 regulated by TGF-β1 is known to target E-Box repressors Zeb1 and Zeb2, which leads to activation of Zeb1/2-regulated genes including collagen type 1 α2, miR-200b/c, miR-217 and miR-216a^18^. The increased miR-200b/c then continue to target Zeb1 and Zeb2 to auto-stimulate their expression^19^. Both miR-217 and miR-216a are known to promote mesangial cell expansion and hypertrophy by silencing PTEN (phosphatase and tenin homolog), the major negative regulator of Akt signaling^20^. Additionally, miR-377 can increase the production of fibronectin through targeting PAK1 and superoxide dismutase^21^. Despite extensive efforts, whether other microRNAs play roles in diabetic glomerulosclerosis remains to be explored and characterized.

The miR-29 family (miR-29a, miR-29b, and miR-29c) is known to directly target extracellular matrix genes and reportedly acts as fibrotic regulators in several tissue types^22^. Cancer-induced liver fibrosis is associated with decreased miR-29a signaling^23^. Moreover, miR-29 mediates the anti-fibrotic activity of hepatocyte growth factor on hepatic stellate cells^24^, and reduces fibrogenesis in dystrophic muscles^25^. Evidence has also shown that loss of miR-29b accelerates, but gain of miR-29b prevents, TGF-β1-mediated renal fibrosis reactions^26^,^27^. The miR-29 family is thought as an important downstream mediator of TGF-β1-mediated fibrogenesis^26^,^27^. Previously, we have demonstrated that hyperglycemia impairs miR-29a expression in podocytes, which leads to acceleration of podocyte injury and decreased expression of nephrin and acetylated nephrin^28^. Overexpression of miR-29a in diabetic mice could efficiently support nephrin levels, podocyte viability and renal function^28^. Due to the fact that most miRNAs are highly pleiotropic and act differentially in different cell types, the detailed function and regulation of miR-29a in the pathogenesis of glomerular fibrosis and inflammation need to be further elucidated.

In the study, we investigated whether miR-29a signaling participates in mesangial fibrosis induced by hyperglycemia, and elucidated the relationship between miR-29a action and the DKK1/Wnt/β-catenin signaling in diabetes-induced renal injury. Our data revealed that high extracellular glucose upregulates profibrotic gene expression accompanied by decreased miR-29a expression and attenuated Wnt/β-catenin signaling in glomerular mesangium of mice or in cultured mesangial cells. Moreover, using gain- and loss-of-function experiments, we showed that miR-29a is an important regulator of DKK1 and Wnt/β-catenin signaling, as well as functions to prevent mesangial cell apoptosis and TGF-β1-mediated fibrosis. The possible regulatory network between miR-29a, DKK1/Wnt/β-catenin signaling and TGF-β1-mediated fibrosis in mesangial cells is discuss in the study.

## Results

### Diabetic conditions increase the expression of DKK1 and profibrotic genes, but reduce miR-29a expression, in renal tissues

**Figure 1:**
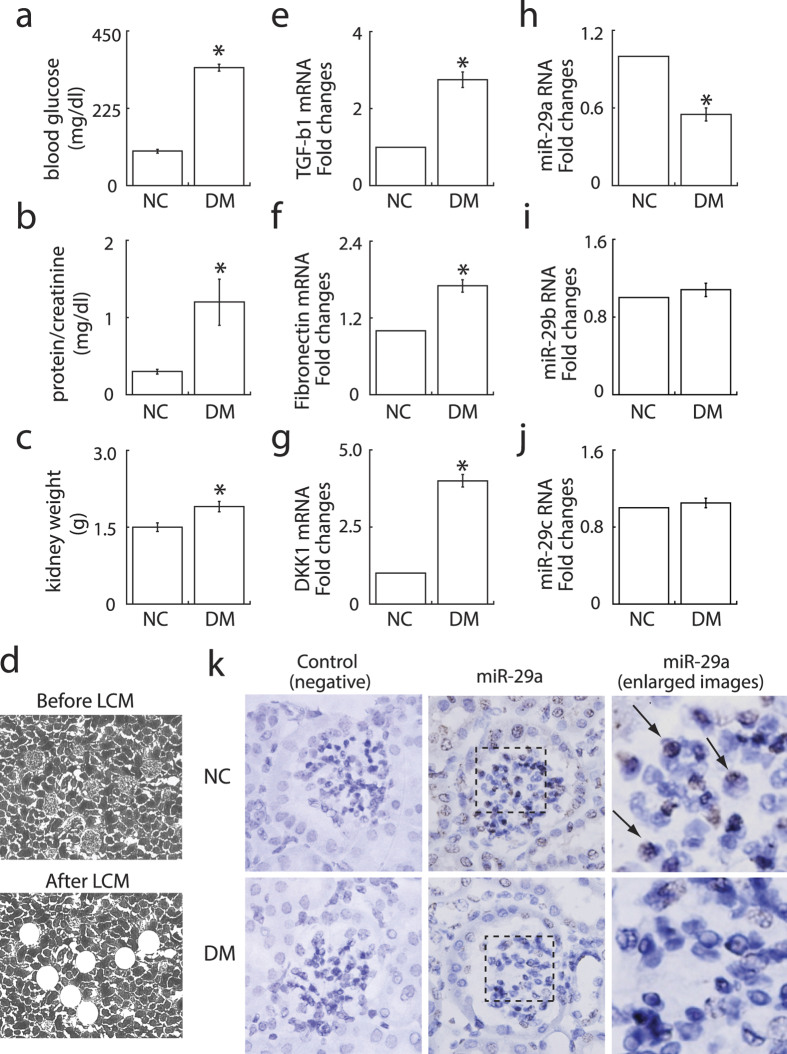
Effects of hyperglycemia on renal function and the expression of miR-29a and profibrotic genes in glomeruli of diabetic kidneys. Diabetic mice (DM; n = 6) displayed increased blood glucose levels (**a**), urinary protein excretion (**b**), and kidney weight (**c**) as compared to normal controls (NC; n = 6). (**d**) Isolation of glomerular compartments in renal tissues by laser-captured microdissection (LCM). Expression levels of TGF-β1 (**e**), fibronectin (**f**), DKK1 (**g**), miR-29a (**h**), miR-29b (**i**) and miR-29c (**j**) in glomeruli of normal and diabetic mice (n = 6 each) were evaluated by quantatative RT-PCR. Experimental results are presented as means ± SEM. *Significant differences (*P* < 0.05) compared with normal controls. (**k**) Representative *in situ* hybridization images of miR-29a in glomeruli of normal and diabetic mice. The box region is enlarged and arrows indicate miR-29a-positive cells (brown-colored cells).

To determine the potential role of miR-29 signaling in glomerular fibrosis induced by diabetes, streptozotocin (STZ)-treated mice were used in the study. After 5-week treatment, STZ-induced diabetic mice showed higher levels of blood glucose concentrations (Fig. 1a) and urinary protein excretion (Fig. 1b), as well as greater kidney weights (Fig. 1c) than their normal controls (Fig. 1, NC). To assess changes in gene expression, glomerular compartments were isolated by laser capture microdissection (Fig. 1d). Quantitative RT-PCR analyses revealed that mRNA levels of TGF-β1 (Fig. 1e), fibronectin (Fig. 1f), and DKK1 (Fig. 1g) were significantly elevated in diabetic glomeruli. By contrast, miR-29a expression (Fig. 1h) was decreased in diabetic glomeruli. There were, however, no significant differences in levels of miR-29b (Fig. 1i) and miR-29c (Fig. 1j) between the normal and diabetic groups. *In situ* hybridization confirmed that mesangial cells within glomeruli in diabetic kidneys displayed lower levels of miR-29a expression as compared to normal controls (Fig. 1k). These results indicated that attenuated miR-29a expression is concomitant with the increased expression of DKK1 and profibrotic factors in diabetic kidneys.

### MiR-29a modulates Wnt/β-catenin signaling activation and prevents renal fibrosis in diabetic mice

**Figure 2:**
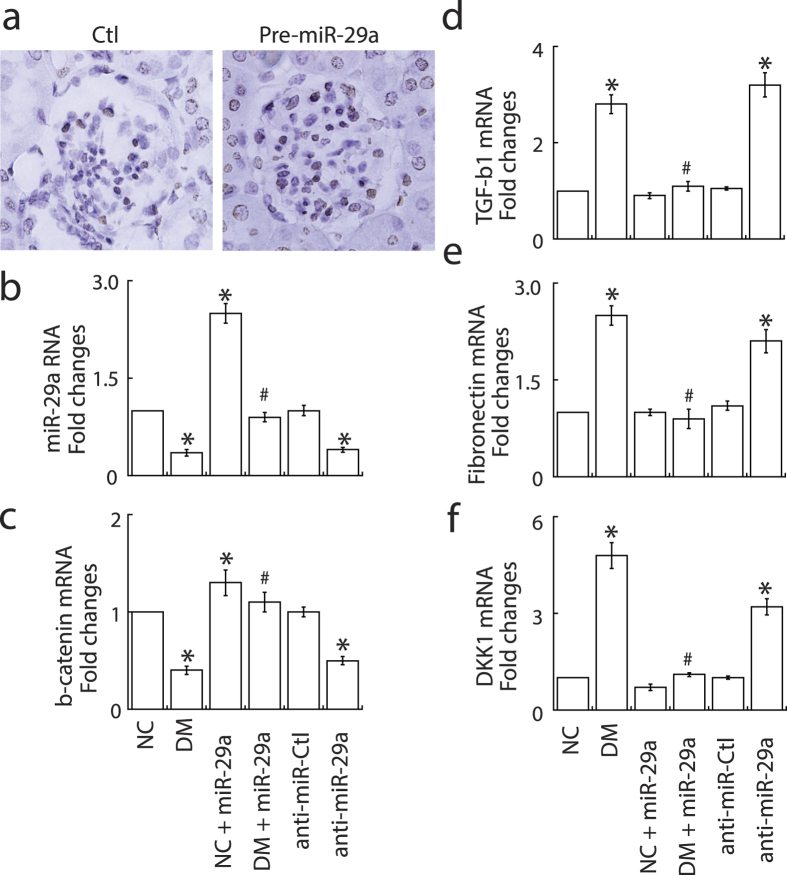
Influence of exogenous miR-29a precursor and miR-29a inhibitor on the expression of profibrotic genes and Wnt/β-catenin signaling components in renal glomeruli. (**a**) *In situ* hybridization of miR-29a in renal tissues of mice that were infected with empty control lentivirus (Ctl) or pre-miR-29-expressing lentivirus (Pre-miR-29a). (**b**–**f**) Changes in expression of glomerular miR-29a, β-catenin, TGF-β1, fibronectin and DKK1 in mice after treatment with STZ (DM; n = 6), pre-miR-29a (NC + miR-29a; n = 6), STZ plus miR-29a precursor (DM + miR-29a; n = 6), a control lentiviral vector (anti-miR-Ctl; n = 6), or miR-29a inhibitor (anti-miR-29a; n = 6). Data are presented as mean ± SEM. NC, normal control; DM, diabetes. Symbol * indicates significant difference vs. NC group and symbol # indicates significant difference vs. DM group (P < 0.05).

**Figure 3:**
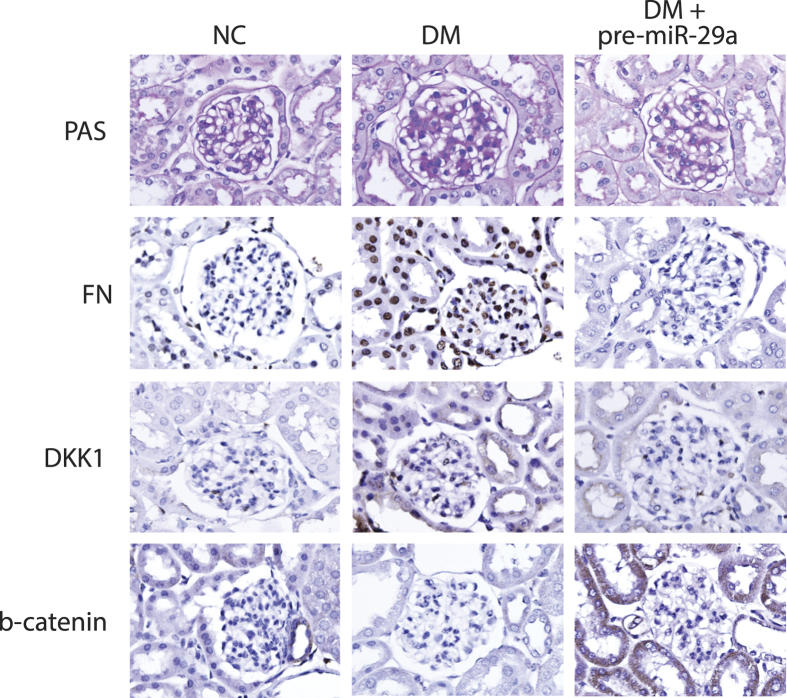
Overexpression of miR-29a precursor in diabetic mice alleviates renal fibrosis and alters Wnt/β-catenin signaling activation in glomerular mesangium. Representative photographs of PAS staining (pink) and immunohistochemical staining (brown) of fibronectin, DKK1 and β-catenin in kidney tissues of normal mice (NC) and diabetic mice (DM) with or without overexpression of miR-29a precursor.

We have previously reported that attenuation of Wnt/β-catenin signaling by DKK1 contributes to renal fibrosis^10^. To investigate whether miR-29a signaling is involved in modulation of Wnt/β-catenin pathway and renal fibrosis, we infected diabetic mice with lentivirus (10^9^ pfu/kg) that encodes miR-29a precursor (pre-miR-29a) to study the association. *In situ* hybridization analysis showed that intense miR-29a signals were specifically detected in renal tissues of pre-miR-29a lentiviral-infected mice (Fig. 2a). Quantitative RT-PCR analysis also confirmed that the level of miR-29a was evidently higher in the pre-miR-29a treatment groups than in the mock treatment groups under normal or diabetic conditions (Fig. 2b). Pre-miR-29a treatment in diabetic mice significantly restored the levels of β-catenin (Fig. 2c), and prevented upregulation of profibrotic factors, including TGF-β1 (Fig. 2d), fibronectin (Fig. 2e), and DKK1 (Fig. 2f), induced by hyperglycemia. Periodic acid-Schiff and immunohistochemical stain also showed that exogenous overexpression of miR-29a in diabetic mice effectively decreased mesangial matrix expansion, and reversed the dysregulated expression of β-catenin, DKK1 and fibronetin in glomeruli (Fig. 3).

**Figure 4:**
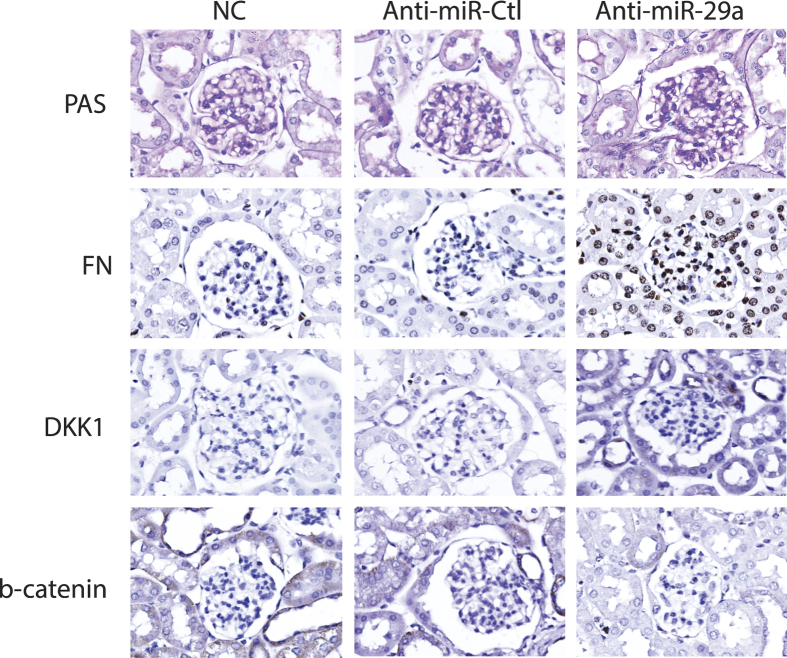
Knockdown of miR-29a in normal mice induces renal fibrosis and downregulates the Wnt/β-catenin signaling. Representative photographs of PAS staining (pink) and immunohistochemical staining (brown) of fibronectin, DKK1 and β-catenin in kidney tissues of normal mice (NC) and normal mice infected with empty control lentivirus (Anti-miR-Ctl) or with lentivirus expressing miR-29a inhibitor (Anti-miR-29a).

On the other hand, lentivirus carrying miR-29a inhibitor (anti-sense miR-29a) was utilized to treat normal mice. Similar to diabetic mice, we found that mice treated with miR-29a inhibitor increased urinary protein excretion (data not shown). In addition, quantitative RT-PCR and immunohistochemical analysis revealed that loss of miR-29a in renal tissues was accompanied by upregulation of profibrotic factors (including TGF-β1, fibronectin and DKK1), and downregulation of β-catenin (Figs 2 and 4). Taken together, these results strongly suggest that miR-29a is involved in the control of specific Wnt/β-catenin signaling components and renal fibrosis.

### MiR-29a transgenic mice are resistant to renal fibrosis after induction of diabetes

**Figure 5:**
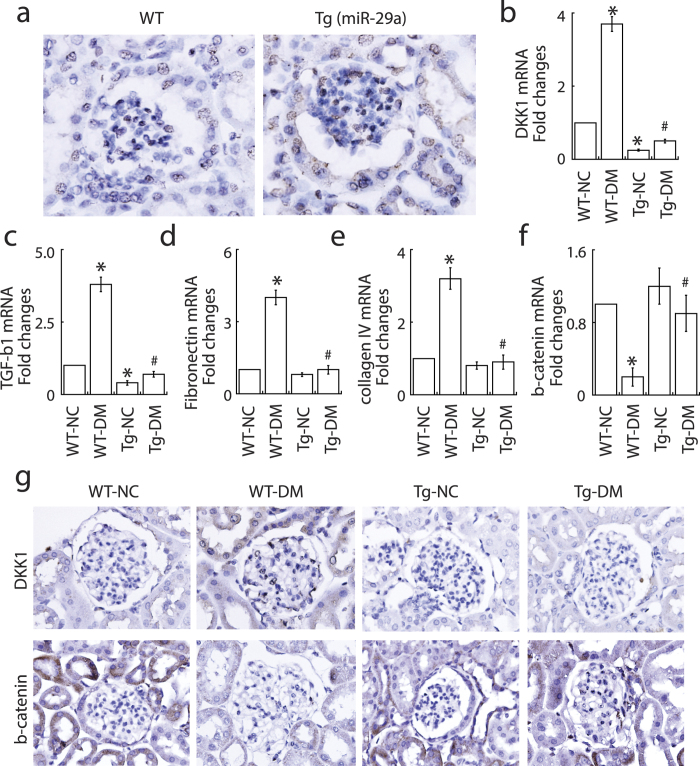
MiR-29a transgenic mice are protected against diabetes-induced renal injury. (**a**) *In situ* hybridization of miR-29a in renal glomeruli of wild-type mice and miR-29a transgenic mice. (**b**–**f**) Expression of DKK1, TGF-β1, fibronectin, collagen IV and β-catenin mRNAs in renal glomeruli of the wild-type (WT) and miR-29a transgenic mice (Tg) after induction of diabetes (n = 6 for each group). NC, normal control; DM, diabetes. Data are expressed as mean ± SEM. Symbol * indicates significant difference vs. wild-type normal (WT-NC) group and symbol # indicates significant difference vs. wild-type diabetic (WT-DM) group (P < 0.05). (**g**) Representative photographs of immunohistochemical staining of DKK1 and β-catenin in glomeruli of wild-type mice and miR-29 transgenic mice without or with STZ treatment.

To further confirm the effect of miR-29a signaling on the modulation of Wnt/β-catenin pathway and renal fibrosis, miR-29a transgenic mice were used in the study (Fig. 5a). Compared to wild-type mice, we found that miR-29a transgenic mice were resistant to develop significant albuminuria following STZ treatment (data not shown). When expression levels of profibrotic factors and β-catenin were evaluated, we consistently found that miR-29a transgenic mice treated with STZ displayed lower levels of DKK1 (Fig. 5b), TGF-β1 (Fig. 5c), fibronectin (Fig. 5d) and collagen IV (Fig. 5e), but higher levels of β-catenin (Fig. 5f), than wild-type STZ-treated mice. High-power field microscopic analysis of glomerular mesangium further demonstrated that miR-29a transgenic mice expressed higher β-catenin and lower DKK1 than wild-type mice after induction of diabetes (Fig. 5g).

### MiR-29a is a positive regulator of Wnt/β-catenin signaling and functions to protect cell apoptosis and fibrosis in renal mesangial cells

**Figure 6:**
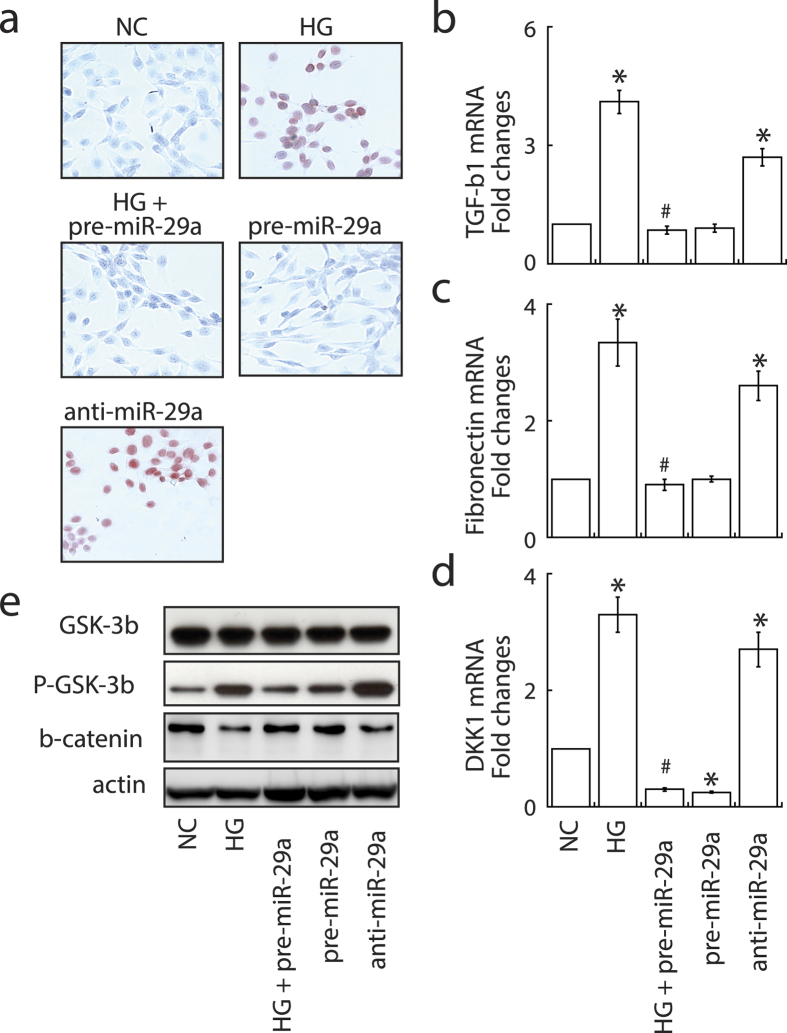
MiR-29a acts a positive regulator of Wnt/β-catenin signaling and functions to prevent apoptosis and fibrogenic activation in cultured renal mesangial cell. (**a**) Effects of high glucose, miR-29a precursor and miR-29a inhibitor on cell apoptosis. Renal mesangial cells were treated with high glucose, miR-29a precursor, miR-29a inhibitor, or a combination of high glucose and miR-29a precursor. TUNEL staining was used to detect apoptotic cells (red). Experiments were performed at least three times and representative results are shown. NC, normal glucose control; HG, high glucose. (**b**–**d**) Changes in the levels of TGF-β1, fibronectin and DKK1 mRNAs in mesangial cells under the above-mentioned conditions. All the experiments were repeated at least three times. Data are indicated as mean ± SEM. Symbol * represents significant difference vs. the normal glucose group, and symbol # represents significant difference vs. the high glucose group (P < 0.05). (**e**) Western blot analysis of GSK-3β phosphorylation and nuclear β-catenin in mesangial cells. Representative blots from three experiments are shown.

Next, we investigated the effect of high glucose, miR-29a precursor or miR-29a inhibitor on cell viability and fibrogenic activation in cultured renal mesangial cells. Microscopic observation showed that mesangial cells cultured in the high-glucose condition or transfected with miR-29a inhibitor strongly induced cell apoptosis as detected by TUNEL staining (Fig. 6a). Treatment of mesangial cells with high glucose or miR-29a inhibitor also promoted the expression of TGF-β1 (Fig. 6b) and fibronectin (Fig. 6c). Importantly, high glucose-induced apoptosis and upregulation of profibrotic genes could be rescued by treatment with miR-29a precursor (Fig. 6a–c). We then investigated whether miR-29a modulates Wnt/β-catenin signaling activation in renal mesangial cells *in vitro*. Treatment of mesangial cells with high glucose or miR-29a inhibitor enhanced levels of DKK1 (Fig. 6d) and GSK-3β phosphorylation (Fig. 6e) accompanied by reduced levels of nuclear β-catenin (Fig. 6e). However, exogenous pre-miR-29a treatment blocked high glucose-induced upregulation of DKK1 and GSK-3β phosphorylation, and downregulation of β-catenin in mesangial cells. These results suggested that miR-29a acts as a positive regulator for the Wnt/β-catenin signaling pathway and modulates cell survival and matrix protein expression in mesangial cells.

### Reciprocal regulation of miR-29a and Wnt signaling in mesangial cells

**Figure 7:**
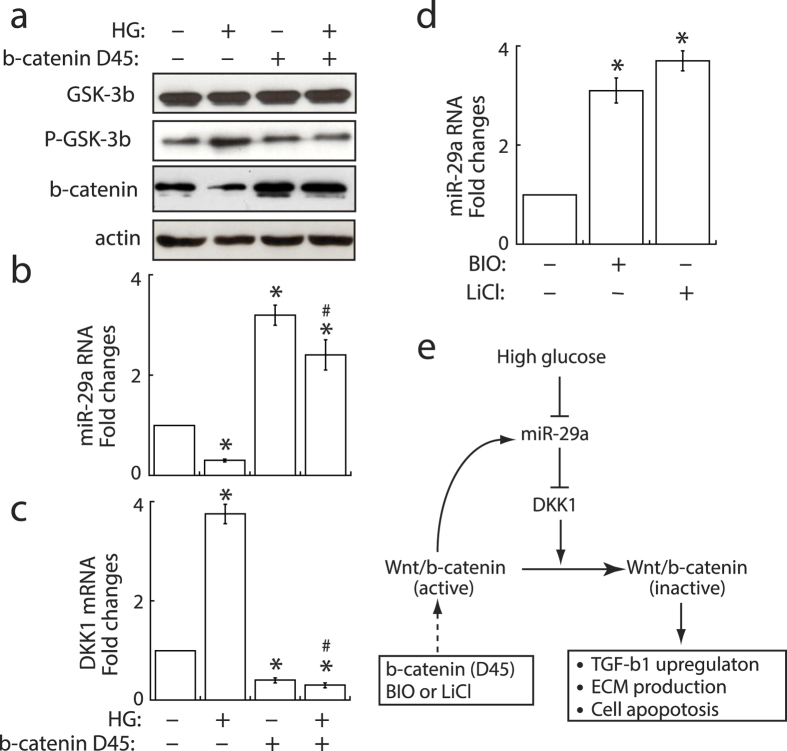
Specific activation of Wnt/β-catanin signaling upregulates miR-29a expression in mesangial cells. (**a**) Western blot analysis of GSK-3β phosphorylation and nuclear β-catenin in mesangial cells that were treated with high glucose (HG) or transfected with β-catenin (Δ45) mutant. Experiments were repeated three times and representative blots are shown. (**b**,**c**) Stabilization of β-catenin by transfecting the β-catenin (Δ45) mutant significantly enhanced miR-29a, but reduced DKK1, in mesangial cells in either normal or high glucose conditions. (**d**) Treatment of mesangial cells with a GSK-3β inhibitor, BIO or LiCL, increased miR-29a expression. All quantitative RT-PCR experiments shown above were independently repeated at least three times. Symbol * indicates significant difference vs. the normal glucose group, and symbol # indicates significant difference vs. the high glucose group (P < 0.05). (**e**) Proposed model for the reciprocal regulation of miR-29a and Wnt/β-catenin signaling.

On the other hand, we analyzed whether Wnt/β-catenin signaling affected the expression of miR-29a in cultured mesangial cells. Stabilization of β-catenin by transfecting the β-catenin (Δ45) mutant (Fig. 7a) in mesangial cells substantially increased (up to 3 fold) the expression of miR-29a (Fig. 7b), suggesting that a positive regulatory circuit exists between miR-29a and Wnt/β-catenin signaling. Transfection with β-catenin (Δ45) mutant also prevented high glucose-induced downregulation of miR-29a (Fig. 7b) and upregulation of DKK1 (Fig. 7c). The reciprocal regulation of miR-29a and Wnt/β-catenin signaling was further demonstrated by incubating mesangial cells with a GSK-3β inhibitor, bromoindirubin-3′-oxime (BIO) or LiCl. Activation of Wnt/β-catenin signaling by BIO or LiCl in mesangial cells evidently increased miR-29a expression (Fig. 7d). The reciprocal regulation of miR-29a action and DKK1/Wnt/β-catenin signaling may be critical for controlling cell survival and the production of extracellular matrix in renal mesangial cells (Fig. 7e).

## Discussion

The miR-29 family members include miR-29a, miR-29b and miR-29c, which share a common seed region sequence^22^. MiR-29s are encoded by two gene clusers, leading to the miR-29b-1/miR-29a and miR-29b-2/miR29c precursors^22^. Differential regulation in miR-29 gene clusters and different subcellular distribution and stability of miR-29 members have suggested that the impact of individual miR-29 member may vary in different biological processes or diseases. Previously, we demonstrated that miR-29a, but not miR-29b and miR-29c, was significantly reduced in renal glomeruli of STZ-treated diabetic mice^28^. In the study, we confirmed our previous observations and extend these findings to further invesitigate the role of miR-29a in diabetes-induced glomerulosclerosis.

In our animal testing, we presented that decreased miR-29a expression and attenuated Wnt/β-catenin signaling (downregulation of β-catenin and upregulation of DKK1) were associated with increased urinary protein excretion and increased expression of profibrotic genes in diabetic kidneys (Figs 1 and 2). *In situ* hybridization confirmed that decreased miR-29a expression was observed in glomerular mesangial cells of diabetic mice (Fig. 1k). Due to low delivery efficiency of chemically synthesized microRNAs in an *in vivo* experiment, we here used a lentivirus-mediated delivery strategy to modulate miR-29a expression in mice. Gain-of-function of miR-29a by using lentiviral expression vector substantially increased levels of β-catenin and inhibited upregulation of profibrotic genes (DKK1, TGF-β1 and fibronectin) in glomeruli of diabetic mice (Figs 2 and 3). In contrast, inhibition of miR-29a in normal mice using lentivirus containing a miR-29a antisense inhibitor promoted the production of profibrotic genes and reduced β-catenin expression (Figs 2 and 4). Although we did not perform a dose-response transduction experiment with lentivirus, our results consistently supported that i) miR-29a modulates both DKK1/Wnt/β-catenin signaling and TGF-β1-mediated fibrosis in renal tissues, and ii) restoration of miR-29a and its downstream signaling is beneficial for improving diabetes-mediated renal fibrosis. Interestingly, we also demonstrated that miR-29a transgenic mice that retain a sustained Wnt/β-catenin signaling activity acquire the ability to protect extracellular matrix accumulation and renal injury after induction of diabetes (Fig. 5). Overall, our findings strongly suggest that exogenous miR-29a precursor gene delivery has therapeutic potential for improving diabetes-mediated renal deterioration. However, to avoid the potential side effect caused by the overexpression of miR-29 in other tissues or organs, the development of lentiviral vectors carrying a kidney-specific miR-29a expression would be required in the future for clinical application.

In addition to glomerular mesangium, we detected extensive staining of β-catenin in tubular epithelium lining tubules, and that this was significantly affected by the gain or loss of miR-29a (Figs 3 and 4). We only focused on mesangial cells in glomerular sclerosis in the present study, and further studies are warranted to elucidate the exact role of miR-29a in tubular epithelial cells under diabetic conditions.

Consistent with *in vivo* experiments, our *in vitro* experiments showed that high glucose-exposed mesangial cells expressed lower levels of miR-29a and nuclear β-catenin, but higher levels of DKK1, GSK-3β phosphorylation, TGF-β1 and fibronectin, than normal mesangial cells (Figs 6 and 7). In addition to fibrogenic activation, high glucose also induced apoptotic cell death in cultured mesangial cells (Fig. 6a). Restoration of miR-29a by expressing miR-29a precursor positively modulated the Wnt/β-catenin signaling activity, and significantly attenuated high glucose-induced fibrosis and apoptosis in renal mesangial cells. Similar to treatment with high glucose, inhibition of miR-29a in mesangial cells sufficiently promoted cell apoptosis and fibrosis (Fig. 6). All of these experiments revealed that miR-29a acts as a positive regulator of the Wnt/β-catenin signaling pathway and as an anti-fibrotic factor. Although miR-29a importantly regulated the activation of Wnt/β-catenin signaling *in vitro* and *in vivo*, we unexpectedly found that miR-29a expression could be reversely controlled by Wnt/β-catenin signaling activation via a positive feedback loop in mesangial cells (Fig. 7e). Our *in vitro* data therefore suggest that a complicated regulatory network may exist between miR-29a, DKK1/Wnt/β-catenin signaling and TGF-β1-mediated fibrosis.

Previous studies have shown that miR-29 members are downstream target genes of TGF-β1, and TGF-β1/Smad3 signaling is required for repression of miR-29 expression during renal fibrosis^26^,^27^. Bioinformatical and experimental analysis have also revealed that miR-29s act as anti-fibrotic factors by directly targeting a large number of extracellular matrix genes^22^,^29^,^30^. In the study, we apparently showed that miR-29a could be an important regulator of TGF-β1 signaling in renal fibrosis. Gain or loss of miR-29a function in animal models or in cultured mesangial cells evidently modulated TGF-β1-mediated fibrosis. Currently, we do not know how miR-29a down-regulates TGF-β1. Due to the fact that i) miR-29a action is associated with the activation of Wnt/β-catenin signaling and ii) active Wnt/β-catenin signaling reportedly represses TGF-β1^9^, it is possible that repression of TGF-β1 signaling by miR-29a may be mediated through the Wnt/β-catenin pathway. However, more work is needed to prove this hypothesis.

We have previously demonstrated that imbalanced expression of DKK1 (a Wnt antagonist) and Wnt signaling components (nuclear β-catenin) contributes to hyperglycemia-mediated cell apoptosis and fibrotic matrix synthesis in diabetic kidneys^9^,^10^. Despite the importance of DKK1/Wnt/β-catenin signaling in renal fibrosis, the upstream regulatory signaling that regulates DKK1 expression and Wnt/β-catenin signaling activation remained unclear. MiR-29a may be a candidate for such a gene in regulating DKK1 expression and Wnt/β-catenin signaling activation in renal cells, Although the relationship between miR-29a and the Wnt/β-catenin signaling activation may vary from cell type to cell type^31^, several studies on osteoblastic cell differentiation have reported that miR-29a could negatively regulate DKK1 expression through direct binding to the 3′-UTR of DKK1 mRNA, which lead to activation of Wnt/β-catenin signaling^32^,^33^,^34^. Consistent with this notion, our data also showed that gain or loss of miR-29a function proportionally altered the expression of DKK1 and β-catenin in both *in vitro* and *in vivo* models of diabetes. We stongly suggest that DKK1 may be a key mediator that links both miR-29a action and the Wnt/β-catenin signaling in renal cells.

Additionally, miR-29a expression may be controlled by Wnt/β-catenin signaling via a positive feedback loop in renal mesangial cells. Specific activation of Wnt/β-catenin signaling by stable β-catenin (Δ45) expression or by BIO or LiCl effectively increased the levels of miR-29a (Fig. 7). Interestingly, two consensus TCF/LEF sites in the miR-29a promoter have been identified previously^32^, which confer responsiveness to Wnt/β-catenin signaling. The reciprocal regulation between miR-29a and Wnt/β-catenin signaling in renal cells may be important for controlling the fibrogenic activation (Fig. 7e).

In conclusion, data from our *in vitro* and *in vivo* experiments have shown that miR-29a is an upstream regulator of DKK1 and Wnt/β-catenin signaling, and functions to protect mesangial cell apoptosis and fibrosis. These findings also implicate that manipulation of miR-29a action may provide a potential therapeutic approach for treating diabetes-induced glomerular sclerosis.

## Materials and Methods

### Diabetic animal models

Four-month-old male FVB mice (BioLasco Biotechnology Co., Taiwan) were intraperitoneally given 50 mg/kg streptozotocin (STZ) to induce diabetes. Each STZ-induced diabetic mouse was given 1–2 unit/kg insulin to equalize blood glucose levels as previously described^9^,^10^. Animals with post fasting blood glucose (200–300 mg/dl) were considered as diabetes. Diabetic or normal animals were sacrificed with an overdose of sodium pentobarbital at 5 weeks (n = 6) after diabetes. All animal experiments were approved by the Institutional Animal Care and Use Committee of Chang Gung Memorial Hospital, and were performed in accordance with the Animal Protection Law by the Council of Agriculture, Executive Yuan (R.O.C.) and the guideline of National Research Council (U.S.A.) for the care and use of laboratory animals.

### Lentiviral delivery of miR-29a precursor and miR-29a inhibitor in a mouse model

Lentiviral-based pMIF-cGFP-zeo and pmiR-ZIP shRNA expression vectors (System Biosciences, Mountain View, CA) were used to drive the expression of miR-29a precursor and miR-29a inhibitor (antisense miR-29a), respectively^28^. The lentiviral-based expression constructs were co-transfected with pPACKF1 vector into 293T cells. Lentiviruses were prepared by CsCl density-gradient ultracentrifugation. Anesthesized mice were given 10^9^ pfu/kg lentivirus suspensions through tail vein injection. In some experiments, mice were given with empty lentiviral vector as a scrambled control. All protocols were performed in P2 level laboratory and infected animals were husbandry in isolated ventilation cages and negative pressure holding rooms. At 8 weeks after injection, animals were sacrificed and renal tissues were dissected for studies.

### MiR-29a transgenic mice

MiR-29a transgenic mice (FVB/miR-29a^Tg^) were generated and maintained as described previously^28^. Briefly, human PGK promoter and human miR-29a precursor full-length sequences were cloned into the pUSE expression vector. The constructed miR-29a-containing DNA fragment was then transferred into fertilized eggs from FVB/N mice. The eggs were further transferred into ICR foster mothers.

### Urine and blood biochemistry

Peripheral blood and urine samples were collected to evaluate renal function in mice. Hemoglobin A1c and blood glucose levels in serum were determined according to the manufacturer’s instructions (Primus Diagnostics, Trinty Biotech Co. Kansas City, MO). Urinary albumin (Dade Behring Inc., Newark NJ) and creatinine (Formosa Biomedical Technology Corp, Taipei, Taiwan) were measured using the respective assay kits. Urinary albumin and protein excretion were normalized to urinary creatinine levels.

### Laser capture microdissection

Laser capture microdissection were performed under RNAase-free conditions as described previously^10^. Renal tissues were longitudinally cut into 4 μm thick. Glomerular mesangium in sections were captured using a VERITAS™ laser-captured dissection system (Arcturus Bioscience Inc., CA) according to manufacturer instructions. Two hundred glomerular mesangium from 6 sections of each animal in each group were dissected for extracting total RNAs. The resultant RNA samples were subjected to quantitative RT-PCR analysis.

### Quantitative reverse transcription-PCR (RT-PCR)

Total RNAs were mixed with *mir*Vana 5 × RT Buffer, 1 × *mir*Vana specific RT primers, and ArrayScript Enzyme Mix (Ambion Inc, Austin), and reversely transcribed into cDNA. Templates were then mixed with a PCR mixture containing specific primers and quantitative PCR was carried out using ABI 7900 Detection System (Applied Biosystems), as described previously^10^,^28^. Specific RT primers and PCR primers for detecting TGF-β1 mRNA, fibornectin mRNA, miR-29a, miR-29b, miR-29c and housekeeping gene 5S were obtained from Ambion Inc. Fold change was calculated as 2^−ΔΔCt^, where ΔΔCt = ΔCt_treatment_ − ΔCt_vehicle_ and ΔCt = Ct_target_ − Ct_5S or β-actin_.

### *In situ* hybridization

Paraffin wax-embedded renal tissues were cut into 5 μm thick sections. A Digoxigenin-labeled locked nucleotide probe for miR-29a (Exiquon Biotechnology Inc) was used. Sections were subjected to de-protein (10 μg/ml Proteinase K at 37 C for 5 min), pre-hybridization (50% formamide, 5 X SSC, 0.1% Tween, 50 μg/ml heparin, 500 μg/ml yeast RNA), and then hybridization (0.3 M NaCl, 20 mM Tris-HCl, 0.5 mM EDTA, 10 mM NaPO4, 10% dextran sulfate, 1X Denhardt, 0.5 mg/ml yeast RNA and digoxigenin-labeled probes). The digoxigenin-labeled transcripts were detected using digoxigenin antibody conjugated horseradish peroxidase, and counterstained with hematoxylin.

### Periodic acid-Schiff (PAS) and immunohistochemical stains

For evaluation of fibrosis matrix, renal tissue sections were subjected to periodic acid-Schiff staining according to manufacturer instructions (Sigma-Aldrich Inc., St Louis, MO). Antibodies against fibronectin (F2372; Bioworld Tech.), DKK1 (sc-25516; Santa Cruz) and β-catenin (#9562; Cell Signaling), as well as horseradish peroxidase-3′-, 3′-diaminobenzidine kits (R & D Systems, Minneapolis, MN) were used for immunohistochemical staining.

### *In vitro* mesangial cell cultures

Mouse SV40 MES-13 glomerular mesangial cells were obtained from American Type Culture Collection, Manassas, VA. Cells (1 × 10^6^ cells/well, six-well plate) were incubated in DMEM medium containing either 5 or 35 mM D-glucose for 72 h. In some experiments, cells were pretreated with 10 μM (2′Z,3′E)-6-bromoindirubin-3′-oxime (BIO) or 10 μM LiCl.

### Transfection of miR-29a precursor or miR-29a inhibitor

Mature miR-29a precursor and miR-29 inhibitor (antisense oligonucleotide) were obtained from Applied Biosystems-Ambio Inc, Austin, TX. A double-stranded RNA oligonucleotide (Pre-miR™ miRNA Precursor Molecules-Negative Control) and an RNA oligonucleotide (Anti-miR™ miRNA inhibitor-Negative Control) were used. Renal mesangial cells (3 × 10^5^ cells/well, 6-well plate) were cultured until 70–80% confluent and transiently transfected with miR-29a precursor, miR-29a antisense oligonucleotide or scrambled control (5–50 pM) using Lipofectamine 2000.

### Terminal deoxynucleotidyl transferase-mediated deoxyuridine triphosphate-biotin nick end-labeling (TUNEL)

Renal mesangial cells that were treated with high glucose, miR-29a precursor or miR-29a inhibitor were harvested, spun (1 × 10^4^ cells) onto glass slides, and then fixed with 70% methanol for investigation of cell apoptosis using *in situ* cell death detection kits (Roche Diagnostics, Mannheim, Germany). Protocols for sample treatment for TUNEL were performed as previously described^9^.

### DNA transfection and Western blotting

The cDNA fragment encoding β-catenin (Δ45) was cloned into pcDNA3.1 (Invitrogen) as mentioned previously^10^. Sub-confluent cultured cells were transfected with 1 μg plasmid using Lipofectamine 2000 (Invitrogen) according to the manufacturer’s instructions. Cells stably transfected with the plasmids were selected in a medium containing 600 μg/ml G418 (Life Technologies, Gaithersburg, MD). Nuclear extracts of mesangial cells were prepared and subjected to Western blot analysis as described previously^8^. Antibodies to β-catenin (#9562; Cell Signaling), GSK-3β (#9315; Cell Signaling) and phsopho-GSK-3β (Ser9) (#9336; Cell Signaling) were purchased commercially.

### Statistical analysis

All values were expressed as means ± standard errors. An independent-sample *t*-test was used to analyze the difference between the normal and diabetes groups. A parametric analysis of variance and Bonferroni post hoc test were used to analyze the difference among normal, diabetic, and miR-29a transgenic mice.

## Additional Information

**How to cite this article**: Hsu, Y.-C. *et al*. Protective effects of miR-29a on diabetic glomerular dysfunction by modulation of DKK1/Wnt/β-catenin signaling. *Sci. Rep.* **6**, 30575; doi: 10.1038/srep30575 (2016).
